# T Follicular Helper Cell Subsets and the Associated Cytokine IL-21 in the Pathogenesis and Therapy of Asthma

**DOI:** 10.3389/fimmu.2019.02918

**Published:** 2019-12-13

**Authors:** Fang Gong, Ting Zheng, Pengcheng Zhou

**Affiliations:** ^1^Department of Laboratory Medicine, Affiliated Hospital of Jiangnan University, Wuxi, China; ^2^Department of Immunology and Infectious Disease, The John Curtin School of Medical Research, The Australian National University, Canberra, ACT, Australia; ^3^Laboratory of Immunology for Environment and Health, Shandong Analysis and Test Center, Qilu University of Technology (Shandong Academy of Sciences), Jinan, China

**Keywords:** T follicular helper (T_FH_) cell, interleukin-21 (IL-21), T follicular regulatory (T_FR_) cell, asthma, immunotherapy

## Abstract

For many decades, T helper 2 (T_H_2) cells have been considered to predominantly regulate the pathogenic manifestations of allergic asthma, such as IgE-mediated sensitization, airway hyperresponsiveness, and eosinophil infiltration. However, recent discoveries have significantly shifted our understanding of asthma from a simple T_H_2 cell-dependent disease to a heterogeneous disease regulated by multiple T cell subsets, including T follicular helper (T_FH_) cells. T_FH_ cells, which are a specialized cell population that provides help to B cells, have attracted intensive attention in the past decade because of their crucial role in regulating antibody response in a broad range of diseases. In particular, T_FH_ cells are essential for IgE antibody class-switching. In this review, we summarize the recent progress regarding the role of T_FH_ cells and their signature cytokine interleukin (IL)-21 in asthma from mouse studies and clinical reports. We further discuss future therapeutic strategies to treat asthma by targeting T_FH_ cells and IL-21.

## Introduction

Asthma, one of the most common chronic and non-infectious diseases, affects around 334 million people worldwide (1). Although the mortality rate associated with asthma has declined remarkably with the regular use of inhaled corticosteroids or oral systemic corticosteroids (2, 3), the overall effectiveness of this therapeutic approach has remained debatable, since 5–25% of asthmatic patients are refractory and show resistance to current corticosteroid-based treatments (4). Concurrently, side effects such as poor immune response to infection and increased risk of osteoporosis are associated with long-term corticosteroid treatment in patients with asthma (5, 6). Therefore, novel treatments that can replace the current steroid-based therapies in a larger cohort of asthma patients and reduce the risk of side effects are urgently needed to not only improve patient outcomes but also reduce the economic burden associated with the management of severe asthma.

Because of their myriad effects on inflammatory responses in the respiratory tract, CD4^+^ T cells have been identified as potent regulators of asthma pathogenesis (7). In this regard, T helper 2 (T_H_2) cells have gradually gained recognition in studies on asthma biology (8, 9). Interleukin (IL)-4, IL-5, and IL-13, which are canonical type 2 cytokines produced by T_H_2 cells, prominently mediate the development of asthma and airway inflammation, manifesting as enhanced IgE-mediated sensitization, airway hyperreactivity (AHR), as well as eosinophil infiltration (1, 10). However, emerging evidence suggests that T follicular helper (T_FH_) cells, rather than T_H_2 cells, predominantly produce IL-4 and IL-21 in B cell follicles and closely regulate IgE class-switching during severe asthma development in both mice and humans (11–15). Therefore, a thorough understanding of T_FH_ cells and their signature cytokine IL-21 is important to fully elucidate the pathogenesis of asthma. In this review, we have summarized recent discoveries related to the role of T_FH_ cells and IL-21 in mouse models and patients with asthma. In addition, we have discussed the therapeutic strategies for asthma that are based on modulation of T_FH_ cells and IL-21, which may potentially be translated into clinical use in the near future.

## Biology of T_FH_ Cells

### Generation and Development of T_FH_ Cells

T cell and B cell interactions, particularly the help provided by T cells to B cells, have been demonstrated for decades (16–19). However, the cellular processes underlying this “help” provided to B cell follicles were not fully understood until a specialized CXCR5-expressing CD4^+^ T cell population, which is uniquely regulated by Bcl-6, was identified (20–22). These cells, termed as T_FH_ cells, can access B cell follicles and regulate the germinal center response (23). Over the past decade, significant progress has been achieved in studying T_FH_ cells. These “help”-providing T follicular cells have been revealed to markedly express inducible co-stimulator molecules (ICOS), programmed cell death 1 (PD-1), and CD40-ligand (CD40L), which are essential for interacting with B cells (24). Moreover, T_FH_ cells produce high amounts of the cytokine IL-21 in the B cell follicles (25, 26).

**Figure 1:**
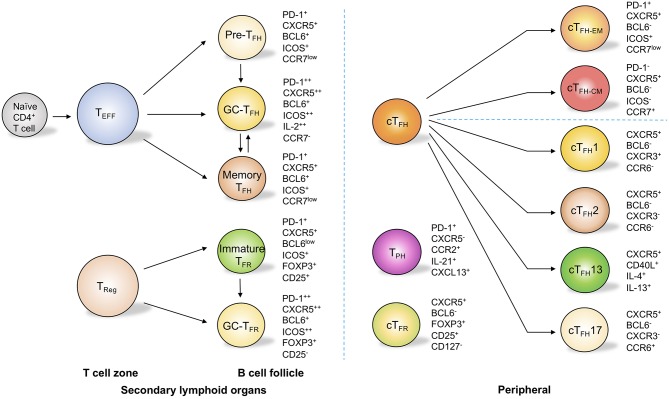
T_FH_ cell and related subsets in secondary lymph organs and in peripheral circulation. In secondary lymphoid organs, T follicular helper (T_FH_) cells are composed of subsets with distinct phenotypes. These subsets include pre-T_FH_ cells, germinal center (GC) T_FH_ cells, and memory T_FH_ cells. Through the upregulation of programmed cell death protein 1 (PD-1) and CXC-chemokine receptor 5 (CXCR5), regulatory T (T_reg_) cells migrate into B cell follicles and become immature T follicular regulatory (T_FR_) cells and germinal center T_FR_ cells. In the peripheral circulation, circulating T_FH_ (cT_FH_) cells can be categorized into effector memory (cT_FH−EM_) cells and central memory (cT_FH−CM_) cells on the basis of the expression of PD-1 and CC-chemokine receptor 7 (CCR7). cT_FH_ cells can also be sub-grouped into cT_FH_1, cT_FH_2, cT_FH_13, and cT_FH_17 cells on the basis of the differential expression of CXCR3 and CCR6 as well as the secretion of interleukin-4 (IL-4) and IL-13. Circulating T_FR_ cells are similar to T_reg_ cells but express CXCR5. Notably, T peripheral helper (T_PH_) cells do not express CXCR5 but can produce IL-21 and CXCL13, which allows them to provide help to B cells.

These molecules are not only determinative of the commitment of T_FH_ cells but are also pivotal for the migration and full functionality of these cells in follicles. After activation by dendritic cells in T cell zone, primed T cells become precursor T_FH_ (pre-T_FH_) cells and downregulate CCR7 and PSGL1 while upregulate CXCR5 for their migration into B cell follicles, where CXCL13, the ligand for CXCR5 is plentifully accumulated (24, 27) (Figure 1). Additionally, EBI2 and PD-1 are critical for the positioning of pre-T_FH_ cells near the T-B border (28, 29). With sustained ICOS stimulation by B cells as well as downregulation of the adhesion molecules EBI2 and S1PR1, T_FH_ cells are allowed to further develop into B cell follicles and are retained in the germinal centers to become germinal center T_FH_ (GC-T_FH_) cells (23, 30, 31) (Figure 1). Bcl-6 is essential for this complex cellular process, since it promotes CXCR5 expression while repressing the expression of the transcription factors T-bet, GATA-3, and RORγt, molecules that are essential for the induction of other subsets of effector CD4^+^ T cells such as T_H_1, T_H_2, and T_H_17 cells (32, 33). Moreover, Bcl-6 inhibits CCR6, PSGL1, CCR7, and S1PR1, the cell surface molecules that regulate non-follicular localization of effector CD4^+^ T cells (23). Antagonistically, the transcriptional repressor B lymphocyte-induced maturation protein 1 (Blimp-1) negatively acts on Bcl-6 to inhibit T_FH_ development. Transcription factors such as c-Maf, FOXO1, Id3, TCF-1, IRF4, and ASCL2 are also known to play important roles in fine-tuning the sophisticated cellular regulatory network during T_FH_ development and function (23).

### Cytokine Milieu Regulates T_FH_ Cell Development and Differentiation

Development of T_FH_ cells is also dependent on the cytokine milieu. Mouse studies have revealed that IL-6, IL-12, and IL-27 induce the expression of Bcl-6 and promote T_FH_ lineage differentiation through the activation of the transcription factors STAT3 and/or STAT4 (34–38). In humans, TGF-β together with IL-12 and IL-23 may contribute to the generation of human T_FH_ cells (39). In contrast, the TGF-β signal exerts suppressive effects in regulating the production of IL-21 and expression of ICOS and Bcl-6 in mouse T_FH_ cells (39). Nevertheless, IL-2 is a suppressive molecule that inhibits the generation of both human and mouse T_FH_ cells in a STAT5- and Blimp1-dependent manner (40, 41).

### Circulating T_FH_ Cells and Subsets of T_FH_ Cells

Although T_FH_ cells possesses distinctive characteristics in comparison with other subsets of CD4^+^ T cells, they can produce T_H_1, T_H_2, and T_H_17-type cytokines. Indeed, Reinhardt et al. (42), Zaretsky and Hirota etc. have shown that T_FH_ cells, especially circulating or tissue-resident T_FH_ cells, produce IL-4 or IL-17 to modulate antibody responses (43, 44). *Bona fide* germinal center T_FH_ cells can also produce IL-4, IFN-γ, or IL-17 to regulate antibody outcomes (42–44).

After the contraction phase of the immune response, a small proportion of CD4^+^ T cells give rise to memory T cells, which confer long-lasting immunity to the host to defend it against recurrent invasions of pathogens. Indeed, MacLeod et al. (45) have shown that CXCR5^+^ memory CD4^+^ T (memory T_FH_) cells (Figure 1) accelerate the generation of functional T_FH_ cells and promote OVA-specific IgG1 titers in OVA immunization. Moreover, influenza vaccination promotes the levels of circulating T_FH_ cells (cT_FH_) cells in human blood, and these cT_FH_ cells correlate with a boosting of antigen-specific B cell response (46). These data strongly suggest that memory T_FH_ cells exist in circulating blood and that these cells can foster rapid and high-quality antibody response.

Interestingly, memory T_FH_ cells in circulation are not only able to promote recall response, but are with plasticity to give rise to other functional effector T cells in different contexts (47, 48). It is also noticed in germinal center that GC-T_FH_ cells switch to produce IL-4 from IL-21 as the germinal center reaction evolved (49). These evidences suggest that T_FH_ cells are not terminally differentiated cells and maintain flexibility to convert into other functional CD4^+^ T cell subsets.

On the basis of the differential expressions of the chemokine receptors CXCR3 and CCR6, peripheral circulating T_FH_ (cT_FH_) cells can be divided into three major subsets: cT_FH_1 cells (BCL6^−^CXCR3^+^CCR6^−^), cT_FH_2 cells (BCL6^−^CXCR3^−^CCR6^−^), and cT_FH_17 (BCL6^−^CXCR3^−^CCR6^+^) cells (50) (Figure 1). These subsets are transcriptionally different and produce distinct cytokines to regulate humoral response (50). Of note, cT_FH_2 and cT_FH_17 cells, but not the cT_FH_1 population, are characterized as efficient helper T_FH_ cells to promote the class-switching of immunoglobulin (50). cT_FH_2 cells promote IgG and IgE secretion, whereas blood cT_FH_17 cells induce IgG and IgA secretion (50). Interestingly, a group of peripheral T cells defined as T peripheral helper cells (T_PH_) do not express CXCR5 but can produce IL-21 and CXCL13 (Figure 1), which allows them to provide help to B cells (51, 52). Meanwhile, a group of CD4^+^ T cells expressing CXCR3 and PD-1 but not CXCR5 have been found in both blood and tubulointerstitial areas in lupus patients (53). These cells provide the help to B cells through the production of IL-10 and succinate instead of IL-21 (53). It is with interest to know in the future how these non-classic “B cell help” CD4^+^ T cells correlate with each other and with classic T_FH_ cells. Notably, classic human circulating T_FH_ cells can also be categorized into distinct effector stages by evaluating the expression levels of ICOS, PD-1, and CCR7 (54, 55). On the basis of this strategy, activated-stage (effector memory) cT_FH_ (cT_FH−EM_) cells are defined as PD-1^+^CXCR5^+^BCL6^−^ICOS^+^CCR7^low^ cells, which are similar to pre-T_FH_ cells, while PD-1^−^CXCR5^+^BCL6^−^ICOS^−^CCR7^+^ cells are characterized as central memory cT_FH_ cells (cT_FH−CM_) and can persist for weeks after antigen stimulation (54, 55) (Figure 1). Interestingly, within blood cT_FH_1 cells, the helper ability is restricted mostly to the activated ICOS^+^PD-1^+^CCR7^low^ subset, while within cT_FH_2 and cT_FH_17 cells, both activated and central memory subsets are capable of providing help signals to the B cells (56, 57). In fact, the activated ICOS^+^PD-1^+^CCR7^low^ subset represents the most efficient helper cells among cT_FH_ cells (56, 57). Beyond this classification, a study using a murine model with dedicator of cytokinesis 8 (Dock8) deficiency revealed a subset of IL-13-producing T_FH_ cells associated with high-affinity IgE production (58) (Figure 1). These “T_FH_13” cells, which are present in both mice and humans, have a unique cytokine profile (IL-13^+^IL-4^+^) and co-express Bcl-6 and GATA-3 (58). These cells were further demonstrated to be responsible for the production of high-affinity anaphylactic IgE but not low-affinity IgE (58).

## Role of T_FH_ Cells in Asthma Pathogenesis

Since T_FH_ cells are indispensable for antibody maturation, investigators have studied the role of these cells in many disease contexts, including asthma (23). Emerging evidence from both mouse and human studies has elucidated that subsets of T_FH_ cells differentially contribute to the development of asthma (Table 1). These observations have broadened our understanding of asthma and provided novel options to treat asthma by targeting T_FH_ cells from different angles.

**Table 1:** T follicular cells in mouse/human asthma and related allergic diseases.

**Species**	**Model/Patients**	**Location**	**Dysfunction**	**Effect**	**References**
Mouse	Peanut	mLN	T_FH_1↑	T_FH_ cells promote peanut-specific IgE production.	(59)
	HDM	mLN	T_FH_2↑	T_FH_ cells are precursors of HDM-specific T_H_2 cells.	(11)
	HDM	mLN	T_FH_2↑	T_FH_ cells amplify T_H_2 cell function in allergic airway inflammation; T_FH_ cells support the sustained production of IgE antibody *in vivo*.	(12–14)
	HDM	Lung	CD4^+^IL-21^+^↑	Promotes local inflammation in the airway	(12, 60, 61)
	HDM and Peanut	mLN	T_FH_13↑	T_FH_ cells are required for production of high-affinity, but not low-affinity, IgE and subsequent allergen-induced anaphylaxis.	(58)
	HDM	mLN	T_FH_13↑, T_FR_ ↓	T_FR_ cells can limit T_FH_13 cell-promoted IgE production.	(62)
	Transplantation (not clear in Asthma)	mLN, Spleen	T_FH_17↑, T_FR_ ↓	IL-10-producing marginal zone precursor B cells control the differentiation of T_FH_ cells and are necessary for immune tolerance.	(63)
	OVA Immunization	mLN, Spleen	T_FR_ ↓	Deficiency of T_FR_ cells leads to excessive humoral immune responses.	(64, 65)
Human	Juvenile Dermatomyositis (not clear in Asthma)	Blood	cT_FH_1↓, cT_FH_2↑, cT_FH_17↑	cT_FH_2 and cT_FH_17 cells, but not cT_FH_1 population, are characterized as efficient helper T_FH_ cells to promote the class-switching of immunoglobulins.	(50)
	Allergic Asthma	Blood	cT_FH_2↑	T_FH_ cells positively correlate with the total IgE level.	(66–68)
	Peanut-Allergen	Blood	cT_FH_13↑	/	(58)
	HDM-Allergen	Blood	cT_FH_↑, cT_FR_ ↓	AIT efficiently modulates the balance of circulating T_FH_ and T_FR_.	(69)
	Allergic Rhinitis	Blood	cT_FR_ ↓	AIT efficiently reinvigorates T_FR_ cells to control IgE production.	(70)
	Asthma	Blood	cT_FR_ ↓	T_FR_ cells produce high amounts of IL-10, which may inhibit the generation of pathogenic T_FH_ cells.	(71–73)
	Rheumatoid Arthritis (not clear in Asthma)	Blood	T_PH_↑	T_PH_ cells promote B cell responses and antibody production.	(51)
	Lupus (not clear in Asthma)	Blood	T_PH_↑	T_PH_ cells stimulate B cell responses via IL-21.	(52)

*HDM, house dust mite; mLN, mediastinal lymph node; AIT, allergen-specific immunotherapy*.

### T_FH_ Cells in Murine Asthma Models

Like in the case of other immune diseases, animal models serve as a feasible approach to investigate the pathogenesis of asthma. To fully understand how T_FH_ cells participate in asthma development, multiple allergens such as house dust mite (HDM), ovalbumin (OVA), molds, and cockroach antigens have been utilized to induce asthma symptoms in mice (74).

Using an HDM-induced asthma mouse model, Ballesteros-Tato et al. (11) showed that the initial intranasal sensitization with HDM directly induces IL-4-producing T_FH_ cells, and these cells then become IL-4^+^IL-13^+^ T_H_2 cells after the HDM challenge. Interestingly, depletion of T_FH_ cells after HDM sensitization successfully prevents T_H_2 cell-mediated immunity after secondary exposure (11). These results are supported by recent studies showing that T_FH_ cells can further differentiate into functional subsets to regulate antibody response (11, 47, 75, 76). Meanwhile, studies have also shown that the airborne allergen HDM independently induces T_H_2 or T_FH_ cells to regulate eosinophilic airway inflammation and IgE production, which raises more questions related to the clear definition of the different roles of T_H_2 and T_FH_ cells in HDM-induced asthma (12, 13). More importantly, these studies have revealed a rare but important IL-21 producing CD4^+^ T cells that are highly pathogenic and can synergize airway inflammation in the lung tissue (12, 60). These cells are different from classical T_FH_ cells as they lack expression of Bcl-6 and CXCR5 and don't require ICOS signaling (12, 60, 61). In another peanut-induced asthma mouse model, T_FH_ cells robustly promoted peanut-specific IgE production (59). In this model, depletion of T_FH_ cells decreased IgE production and protected mice from anaphylaxis without affecting T_H_2 cells (59). Thus, T_FH_ cells are necessary and sufficient for the B cell class-switching and sustained IgE production in the absence of T_H_2 cells (13, 59). In line with this result, mice with T cell specific IL-6R deficiency exhibit limited T_FH_ expansion after HDM sensitization and significantly impaired IgE response (14). Moreover, a rare population defined as IL-13 producing T_FH_ (T_FH_13) cells is reported to be essential for the production of high-affinity IgE antibody and the subsequent allergen-induced anaphylaxis (58). Eliminating T_FH_13 cells or T_FH_ cell-derived IL-13 during allergen immunization results in the abrogation of high-affinity anaphylactic IgE (58).

### T_FH_ Cells in Human Asthma

In human studies, our group and other groups have found significantly higher circulating T_FH_ cell (CXCR5^+^CD4^+^) levels in both child and adult asthma patients in comparison with healthy cohorts (66, 67). Additionally, a skewed peripheral T_FH_ cell phenotype toward the T_FH_2 phenotype has been identified in asthma patients, where the frequency of T_FH_2 cells positively correlated with total IgE levels in the blood (66). We have further observed that circulating T_FH_ cells enhance IgE production, which can be reduced by blocking IL-4 or IL-21 (77). Moreover, the levels of IL-4^+^IL-21^+^CXCR5^+^CD4^+^ T cells have been shown to positively correlate with the total IgE level *in vivo* (77). These results indicate that circulating CXCR5^+^CD4^+^ T_FH_ cells support the germinal center production of IgE in asthma patients. Interestingly, studies using microRNA have revealed that miR-192 is a promising therapeutic target in asthma patients as it inhibits T_FH_ cell differentiation (67, 78). Of note, allergen-specific immunotherapy (AIT), which leads to improved prognosis in allergic patients, efficiently reduces circulating T_FH_ cell levels (68, 69). AIT treatment also markedly increases the frequency of T follicular regulatory (T_FR_) cells, which are known to suppress the germinal center reaction (69, 70).

## Biology of IL-21

IL-21 and IL-21R were discovered in 2000 (79, 80). As a pleiotropic type I four-α-helical bundle cytokine, IL-21 is predominantly produced by NKT cells and activated CD4^+^ T cells such as T_H_9 cells, T_H_17 cells, and T_FH_ cells (81, 82). IL-21 exerts its biological function via binding to its heterodimeric receptor. This receptor is composed of the common γ-chain subunit shared with IL-2 family cytokines, including IL-4, IL-7, IL-9, and IL-15, and its own unique receptor (designated IL-21R), a member of the class I cytokine receptor family (83). Although the production of IL-21 is restricted to lymphocytes, IL-21R is universally expressed on a large range of immune and non-immune cells, indicating its broad physiological effects (79, 80). Recent advances have revealed that IL-21 promotes the activation and cytotoxic function of NK and NKT cells (84, 85). IL-21 also enhances the anti-viral and tumor function of CD8^+^ T cells (82, 86, 87). In particular, IL-21 regulates the formation and function of CD4^+^ T cell subsets, including the promotion of IL-17-producing T cells (T_H_17) (88, 89), efficient development of T_FH_ cells (90), and limitation of T_FR_ cells (64). IL-21 is essential for B cell differentiation and activation. In this context, IL-21 induces B cell proliferation and differentiation to either memory B cells or terminally differentiated plasma cells egressing from the germinal center (82, 91). In addition, IL-21 plays fundamental roles in regulating Ig class-switching and maintaining germinal center reaction (82, 92, 93).

**Figure 2:**
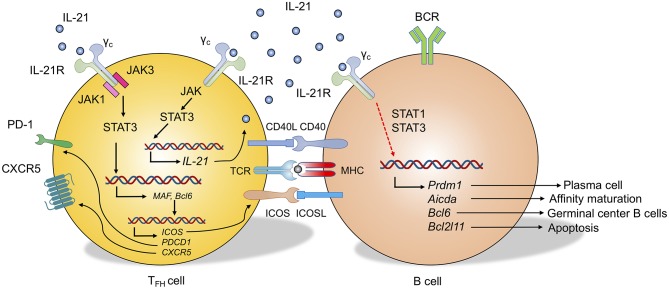
Role of IL-21 in T_FH_ cell and B cell differentiation. IL-21 binds to the IL-21 receptor (IL-21R), which dimerizes with the common cytokine receptor-γ chain (γ_c_) to form the IL-21R complex. In T_FH_ cells, IL-21 signaling activates Janus kinase 1 (JAK1) and JAK3 to induce phosphorylation of signal transducer and activator of transcription 3 (STAT3, also STAT1 and STAT5 to some extent). STAT3 protein translocates into the cell nucleus and regulates the transcription of target genes, including *IL-21, Maf*, and *Bcl6*. This regulation leads to the autocrine IL-21 by T_FH_ cells and the transcriptional program that upregulates genes encoding CXCR5, ICOS, and PD-1. In B cells, at least partially through STAT1 and STAT3, IL-21 signaling can regulate the gene expression of *Prdm1* (Plasma cell differentiation), *Acida* (Affinity maturation), *Bcl6* (Germinal center B cells), and *Bcl2l11* (B cell apoptosis) which leads to the differentiation of B cells to multiple directions.

As a potent cellular modulator, IL-21 binds to the IL-21R and stabilizes the IL-21R-γ_c_ (common cytokine receptor γ chain) complex, which leads to the activation of downstream signaling cascades (94). Which signaling pathways are particularly important to regulate the formation, function, and fate of T and B cells? Janus kinase 1 (JAK1) and JAK3 have been shown to be largely activated by the IL-21R-γ_c_ complex. This activation leads to strong phosphorylation of signal transducer and activator of transcription protein 3 (STAT3), which will further dimerize and translocate into the nucleus for target genes (94). In T cells, activated STAT3 signaling results in increased expression of retinoic acid receptor-related orphan receptor-γt (*RORC*) and enhanced production of IL-17 and IL-21 (88, 89, 95, 96). This IL-21-STAT3 axis can also directly promote IL-6 mediated Bcl-6 expression, which induces the upregulation of CXCR5, ICOS, and PD-1 during T_FH_ cell development (23, 25, 97) (Figure 2). Although future studies are required to determine whether IL-21 is superior to other STAT3 inducing cytokines such as IL-6 on the regulation of T_FH_ cells *in vivo* (98), IL-21 is at least partially required for the potentiation of T_FH_-like cells *in vitro* (90, 99). Additionally, IL-21 also regulates the target genes in T cells through BATF, JUN, and IRF4 (100). In B cells, IL-21 maintains Bcl-6 expression in germinal center B cells (101, 102) while it increases the expression of Blimp1 (*Prdm1*), which promotes plasma cell differentiation (91). IL-21 also regulates the apoptosis of B cells through the modulation of BIM (Bcl-2 interacting mediator of cell death) (103, 104) (Figure 2).

These IL-21-initiated pivotal signaling pathways can be targeted through agonists or antagonists (inhibitors) to modulate T and B cell development and function, and more importantly, intervene and treat multiple immune related diseases, including asthma.

## Role of IL-21 in Asthma Pathogenesis

### IL-21 in the Pathogenesis of Murine Asthma

The IL-21 transcript is upregulated in the lung and lung-draining lymph nodes during allergic airway response (12, 105). The protein levels of IL-21 and IL-21R are also increased in the pulmonary tissues of asthmatic rats (106). Additionally, IL-21 level is elevated in the serum and bronchoalveolar lavage fluid (BALF) of asthmatic mice (107, 108).

IL-21 has anti-IgE and anti-inflammatory effects (93, 109–113). Indeed, *Il21r*-deficient mice exhibit high levels of IgE, and IL-21 inhibits IL-4-induced IgE secretion by B cells (105, 114). In the OVA-induced asthma model, the administration of exogenous IL-21 reduced IgE production and decreased eosinophil recruitment into the airway (109). Consistent with this data, Lin et al. have confirmed *in vivo* that intranasal administration of IL-21-expressing adenovirus suppresses allergic responses (115). Additionally, in this model, administration of IL-21 not only reduces the frequency of T_H_2 cells but suppresses the secretion of T_H_2-associated cytokines such as IL-4, IL-5, and IL-13 (115). In line with this observation, Wu et al. have shown that nasal administration of rmIL-21 significantly reduced the AHR, inflammatory cell infiltration, IgE-producing B cell level, and total serum IgE level (116). As mentioned above, serum total and HDM-specific IgE antibody titers are markedly higher in *Il21r*-deficient mice (105). However, *Il21r*-deficient mice develop unexpectedly less AHR in an HDM model of asthma (105). Similar results showing a decline in AHR are also observed in an OVA-induced experimental asthma model using *Il21r*-deficient mice (114). These findings suggest that IL-21 is importantly involved in the development of asthma. While the mechanisms underlying the dichotomy in the role of IL-21 in regulating IgE and AHR remain poorly understood, they are presumably related to the location and timing of the differential accumulation of IL-21 during disease development.

### IL-21 in Human Asthma

The main obstacle in studying the immunopathology of asthma in human subjects is the relative inaccessibility of inflamed tissues. Nonetheless, using bronchial biopsies, IL-21 expression has been shown to be elevated in both moderate and severely asthmatic individuals (105). Additionally, an increased IL-21 level appears to be associated with increased infiltration of inflammatory cells in the submucosa and is correlated with asthma severity (105). In addition, the plasma level of IL-21 is significantly elevated in asthma patients in comparison with healthy controls (117). Consistently, an increased frequency of IL-21-expressing CD4^+^ T cells is also observed in asthma patients. This increased frequency positively correlates with total IgE levels in the blood (77). Moreover, *in vitro* experiments have demonstrated that blocking IL-21 in the coculture assay of B cells with CXCR5^+^CD4^+^ T cells results in decreased IgE antibody production by B cells (77). Besides, Chatterjee et al. have reported that the exon-3 polymorphism C5250T of the IL-21 gene was significantly associated with atopic asthma and total IgE level (118).

## Clinical Implications

The increasing number of studies on T_FH_ cells and IL-21 have inspired numerous possibilities for the development novel immunotherapies to treat asthma. As mentioned above, modulating IL-21 and T_FH_ cell-regulated IgE production may effectively control asthma development and alleviate inflammatory and hyperresponsiveness symptoms in patients.

### T_FH_ Cells and Serum IL-21 as Biomarkers in Asthma

Precise and early diagnosis of asthma and related syndromes is critical for the prompt control of disease development in patients. Lung function tests for timely and accurate diagnosis of asthma are not as feasible in children as they are in adults. As evidenced in recent clinical studies, the frequency of cT_FH_ cells and/or the IL-21 level in peripheral blood mononuclear cells (PBMCs) appear to be the promising diagnostic biomarkers for IgE production and asthma symptoms (66, 67, 77). cT_FH_ cells and IL-21 levels can be potentially included in future diagnostic criteria for asthma. Moreover, future portable devices equipped with a method to analyze cT_FH_ cells and IL-21 may allow efficient and precise diagnosis of asthma in those who have a family history of the disease or are highly susceptible to severe asthma due to genetic defects and environmental factors.

### Limiting Pathogenic T_FH_ Cells in Asthma

Many approaches can be utilized to target pathogenic T_FH_ cells. For example, T_reg_ cells are known to reinstate immune tolerance and prevent exaggerated immune response through their immune-suppressive function (119). Deficiency of T_reg_ cells in both mice and humans leads to the excessive humoral immune responses characterized by spontaneous germinal center formation and increased frequency of pathogenic T_FH_ cells (65, 120, 121). Indeed, temporary depletion of T_reg_ cells leads to enhanced secondary immune response upon antigen re-challenge (65). This enhanced memory immune response occurs partially through the reduction of CTLA-4-directed inhibition of CD80/CD86 on B cells, which results in an increased frequency of T_FH_ cells (65).

Furthermore, by upregulating CXCR5, a significant proportion of T_reg_ cells migrate into B cell follicles and exert suppressive functions on T_FH_ cells and GC B cells (122, 123). These cells, which are termed as T_FR_ cells, are considered to control autoimmunity and germinal center reaction (124) (Figure 1) as well as autoreactive B cell clones in infection (125). Human clinical studies have shown that allergen immunotherapy reinvigorates the T_FR_ cells in patients with allergic rhinitis, and the addition of human T_FR_ cells in the T_FH_ and B cell coculture system remarkably reduced T_FH_ cell-promoted IgE production (70). It is thus of interest to see how T_FR_ cells respond in asthma patients in future studies. Moreover, specialized human IL-10-producing CD25^+^Foxp3^−^ T_FR_ cells effectively control IgE production (126). In the most recent study, Clement et al. revealed that T_FR_ cells can limit T_FH_13 cell-promoted IgE in mouse, and depletion of T_FR_ cells enhances antigen-specific IgE antibody and exacerbates lung inflammation (62). These studies suggest promising paths to inhibit pathogenic T_FH_ cells and IgE production in asthma, and also shed light on the development of novel immunotherapies in asthma patients by promoting T_reg_/T_FR_ cell-mediated suppression of T_FH_ cells.

Administration of cytokine and/or antibodies has been considered to be an effective method to reinstate the balance of immune response in many types of diseases including asthma (127). IL-2 has been shown to vigorously suppress T_FH_ cells (40). Indeed, clinical studies have proven that low-dose IL-2 treatment in systemic lupus erythematosus (SLE) patients safely and effectively limits autoimmunity partially through direct inhibition of self-reactive T_FH_ cells (41). Besides, other cytokines may also potentiate the repression of pathogenic T_FH_ cells in asthma. For example, IL-7 is reported to repress Bcl-6 and the gene profile of T_FH_ cells in chronic viral infection, which leads to the generation of a memory pool of effector T cells (128). Although lack of CXCR5 and Bcl-6 expression, a specialized IL-21 producing CD4^+^ T cell population is reported to provide help to B cells and synergize airway inflammation in lung tissue (12, 60). The role of these cells in human asthma is still unknown, nevertheless, it is of great interest to understand these non-classic T_FH_ cells in the future as targeting on their IL-21 production may ameliorate lung inflammation in asthma. Moreover, IL-10 resolves the inflammation in asthma (71–73). Studies have shown that IL-10-producing marginal zone precursor B cells control the differentiation of T_FH_ cells and are necessary for immune tolerance (63). T_reg_ cells and T_FR_ cells produce high amounts of IL-10, which may be the underlying mechanism of the T_reg_/T_FR_ cell-mediated inhibition of pathogenic T_FH_ cells and allergen-specific IgE antibody production. Type I interferon counteracts with STAT3 to restrain T_FH_ cells (129). Interestingly, type I interferon has been also shown to suppress infection-induced asthma (130, 131). In particular, future studies should aim to determine whether targeting of type I interferons will eliminate pathogenic T_FH_ cells and resolve asthma in patients. Of note, combination therapy with mixed cytokines, cytokine-cells, and cytokine-chemical may provide even better suppression of pathogenic T_FH_ cells. For example, the combination of IL-10 or IL-2 with T_reg_ or T_FR_ cells may synergize the immuno-suppressive function of pathogenic T_FH_ cells and confer improved control of asthma symptoms in patients.

### Modulating IL-21 Signaling in Asthma

IL-21 and IL-21R are emerging as promising targets for novel cytokine-based immunotherapies in many diseases, including SLE, primary immunodeficiency (PID), chronic lymphocytic leukemia (CLL), multiple myeloma (MM), and lymphoma (132–134). Phase I and phase II clinical trials have tested the efficacy and safety of IL-21 administration in limiting malignant melanoma (135–138). These studies provide evidence for the use of IL-21 as a safe and effective immunotherapeutic agent in a broad range of diseases. Because of IL-21's profound effects in controlling IgE production, supplementation of IL-21 may be useful to rebalance the elevated IgE level in asthma (93). It is possible that IL-21 may have multiple roles in asthma, wherein it may sustain germinal center reaction while limiting Ig class-switching toward IgE. This dichotomy in the effects of IL-21 in asthma may be due to the timing and location at which IL-21 is preferentially accumulated. Nevertheless, it is worthwhile to point out that IL-21 administration in other allergic mouse models, including skin allergy, allergic rhinitis, and anaphylaxis, impressively reduces allergen-specific IgE production (111–113). Again, these points of evidence provide confidence for the development and assessment of IL-21-based immunotherapy in allergic asthma.

On the other hand, amelioration of disease symptoms and improved health were observed after delivery of IL-21 neutralizing antibodies or IL-21R blockade in mice in multiple autoimmune and inflammatory disease models, including SLE (139), arthritis (140), graft-vs.-host disease (GVHD) (141, 142), and Crohn's Disease (143). Although it is still not very clear why IL-21 and IL-21R signaling play different roles in asthma, it will be very exciting to see more studies provide definitive evidence on IL-21's immunomodulating functions in regulating T_FH_ cells, IgE production, and germinal center response in asthma.

## Conclusion

In our review of the research using animal models and human patient samples, T_FH_ cell and its signature cytokine IL-21 were evidenced to be largely involved in asthma. In particular, specialized subsets of T_FH_ cells, such as T_FH_2 cells, T_FH_13 cells, and T_FR_ cells closely regulate IgE production in asthma. Future studies using single-cell technology can help us better understand this heterogeneity of the T_FH_ cell population in asthma patients and healthy cohorts. Future studies are also required to elucidate the connection between IL-21 and different subsets of T_FH_ cells as well as T_FR_ cells, and to determine how can we use this follicular regulatory network to control asthma disease. It also remains to be seen how T_FH_, T_FR_ cells and IL-21 are used to better classify the asthma patients, which may help clinicians design personalized and precise medicine for different individuals with asthma.

## Author Contributions

PZ, FG, and TZ wrote the manuscript. PZ revised the manuscript and led the submission.

### Conflict of Interest

The authors declare that the research was conducted in the absence of any commercial or financial relationships that could be construed as a potential conflict of interest.
